# How Neural Crest Transcription Factors Contribute to Melanoma Heterogeneity, Cellular Plasticity, and Treatment Resistance

**DOI:** 10.3390/ijms22115761

**Published:** 2021-05-28

**Authors:** Anja Wessely, Theresa Steeb, Carola Berking, Markus Vincent Heppt

**Affiliations:** 1 Department of Dermatology, Universitätsklinikum Erlangen, Friedrich-Alexander-University Erlangen-Nürnberg (FAU), 91054 Erlangen, Germany; anja.wessely@uk-erlangen.de (A.W.); theresa.steeb@uk-erlangen.de (T.S.); carola.berking@uk-erlangen.de (C.B.); 2 Comprehensive Cancer Center Erlangen-European Metropolitan Area of Nuremberg (CCC ER-EMN), 91054 Erlangen, Germany

**Keywords:** melanoma, neural crest, MITF, MSX1, SOX10, PAX3, FOXD3, cellular plasticity, phenotype switch

## Abstract

Cutaneous melanoma represents one of the deadliest types of skin cancer. The prognosis strongly depends on the disease stage, thus early detection is crucial. New therapies, including BRAF and MEK inhibitors and immunotherapies, have significantly improved the survival of patients in the last decade. However, intrinsic and acquired resistance is still a challenge. In this review, we discuss two major aspects that contribute to the aggressiveness of melanoma, namely, the embryonic origin of melanocytes and melanoma cells and cellular plasticity. First, we summarize the physiological function of epidermal melanocytes and their development from precursor cells that originate from the neural crest (NC). Next, we discuss the concepts of intratumoral heterogeneity, cellular plasticity, and phenotype switching that enable melanoma to adapt to changes in the tumor microenvironment and promote disease progression and drug resistance. Finally, we further dissect the connection of these two aspects by focusing on the transcriptional regulators MSX1, MITF, SOX10, PAX3, and FOXD3. These factors play a key role in NC initiation, NC cell migration, and melanocyte formation, and we discuss how they contribute to cellular plasticity and drug resistance in melanoma.

## 1. Introduction

Cutaneous melanoma represents one of the deadliest types of skin cancer globally with 55,500 deaths annually [1]. In the last 50 years, the incidence has steadily been rising in most Western countries with fair-skinned populations [1]. In 2018, approximately 287,000 new cases were diagnosed worldwide [2], with the highest age-adjusted rates found in New Zealand and Australia [3]. Several risk factors, including intermittent sun exposure; childhood sunburns; fair skin; inability to tan; indoor tanning; presence of a high number of benign nevi; dysplastic nevi; and mutations affecting tumor suppressor genes like *CDK4*, *CDKN2A*, and *RB1* have been identified [4,5]. Besides reducing exposure to ultraviolet radiation through sun protection measures, early detection of suspicious skin lesions represents a key strategy of secondary prevention [6].

Surgical excision of the tumor is the preferred treatment option for most patients. The prognosis strongly depends on the stage of the disease, underlining that early detection of melanoma is of paramount importance for a good prognosis. Currently, five-year overall survival (OS) rates of stage I melanoma patients are almost 100% [7]. On the other side, the disease quickly becomes life-threatening once it metastasizes. Five-year OS rates are only around 20% for patients with stage IV melanoma [8]. However, new systemic therapy approaches targeting immune checkpoint blocking antibodies and small molecule-inhibitors targeting mutant BRAF kinase and its downstream target MEK have drastically changed the landscape of melanoma therapy in the last decade. Indeed, these treatment options seem to significantly improve the survival of patients with advanced melanoma as recently shown [9,10,11]. In summary, they have substantially contributed to an expansion of the treatment spectrum. However, drug resistance is not uncommon and poses a major challenge for long-term survival [1].

## 2. Lessons Learned from Melanoma’s Cells of Origin and Their Embryonic Origin

Why is melanoma so aggressive, and why is its treatment still challenging despite the pioneering advancements achieved within the last decade? The answers to these questions lie at least partly in its cells of origin and their development during embryogenesis. Melanoma arises from melanocytes, the pigment-producing cells located in the basal layer of the epidermis [12]. Melanocytes have specialized organelles termed melanosomes, which contain two types of pigments, the yellow-to-red pheomelanin and the brown-to-black eumelanin [13]. These pigments are allocated by one melanocyte to about 30 surrounding keratinocytes [14], which then wrap it around their nuclei to prevent UV-induced DNA damage [12]. However, eumelanin is the main photoprotective mediator, whereas pheomelanin can be degraded by UV radiation, resulting in the generation of reactive oxygen species that can cause additional DNA damage [15,16]. Melanin formation (melanogenesis) can be stimulated in a para-, auto-, or intracrine fashion [17]. Eumelanin production is promoted via binding of alpha melanocyte-stimulating hormone (α-MSH) to the melanocortin-1 receptor (MC1R), leading to an increase in intracellular cyclic adenosine monophosphate (cAMP) levels and activation of cAMP-response-element-binding protein (CREB) [15,18,19]. Together with the transcription factors sex-determining region Y-box 10 (SOX10) and paired box 3 (PAX3), this induces the transcription of microphthalmia-associated transcription factor (MITF), which upregulates the expression of enzymes that are required for the various steps of melanogenesis originating from the amino acid tryptophan [13,15,18]. Therefore, MITF, which is a member of the basic helix-loop-helix leucine zipper family of transcription factors [20], is often referred to as the “master regulator” of melanogenesis. MITF also plays a key role in the development of melanocytes and will be discussed in the following review. 

Although melanocytes reside in the skin like keratinocytes, their embryonic origin differs significantly. The embryonic origin of melanocytic precursor cells lies in the neural crest (NC) [21]. This transient accumulation of multipotent precursor cells is located between the neural tube and the ectoderm, and also gives rise to other cell types, such as neurons and glial cells, smooth muscle cells of the heart, adipocytes, and secretory adrenal cells [21]. The development of the neural crest is tightly linked to neurulation, the formation of the neural tube, which occurs already at an early stage of embryogenesis. Within the first weeks after fertilization, neuroectodermal cells start to form the neural plate, which subsequently folds at approximately day 18 and finally closes to form the neural tube that starts at day 21 and finishes around day 26 to 28 post-fertilization in humans [22]. The cells forming the NC originate from the borders of the neural plate and form the dorsal part of the neural tube [23,24]. Giving rise to various cell types that are located in distinct organs and body sites can only be achieved by a high migratory capacity of NC cells [23]. Regarding the melanocytic lineage, immature precursor cells migrate along the dorsolateral “route” between the ectoderm and the somites towards their final location in the epidermis [21,25]. This requires a switch from an epithelial to a more migratory, mesenchymal phenotype in a process termed delamination [23]. Additionally, some studies indicate that a subpopulation of melanocytes originates from precursor cells that have migrated on the ventral “route” [21,25,26,27]. During the migration through the dermis, the melanocytic precursor cells now termed melanoblast proliferate, cross the basement membrane, and finally arrive in the basal layer of the epidermis, their final location where they differentiate to melanocytes and switch back to an epithelial, non-migratory phenotype again [28]. 

**Figure 1:**
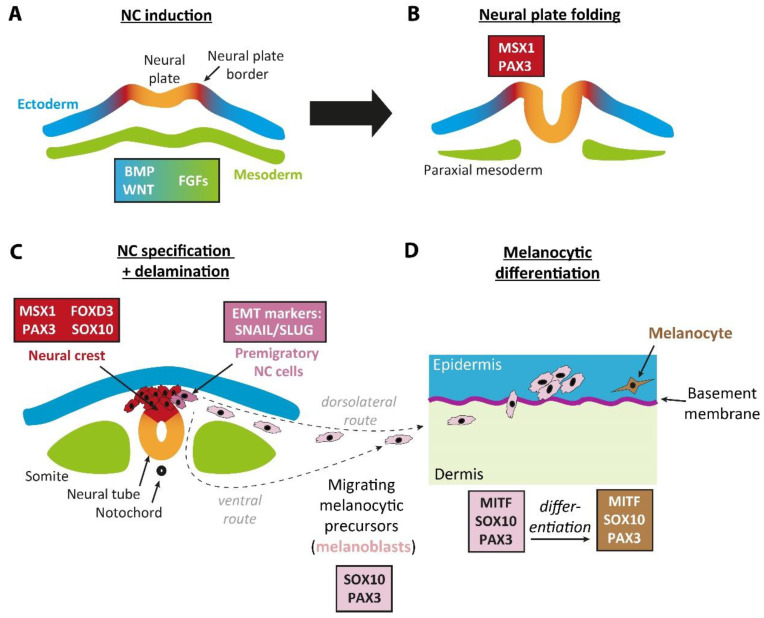
MSX1, FOXD3, SOX10, PAX3, and MITF expression in melanocytic development. (**A**,**B**) During early embryogenesis, the first phases of neurulation and neural plate folding occur. BMP and WNT secreted by the non-neural ectoderm (blue) and FGFs secreted by mesodermal cells (green) induce MSX1 and PAX3 expression in cells located at the neural plate border (red). These cells later give rise to the NC. (**C**) After the neural plate folding, NC cells (red) form the dorsal part of the neural tube (orange) and express NC-specific markers including MSX1, PAX3, FOXD3, and SOX10. MSX1 also triggers delamination, a process leading to an epithelial-to-mesenchymal transition (EMT) of NC cells that is characterized by an increased expression of transcription factors like SNAIL and SLUG and a switch of cadherin expression. Consequently, multipotent NC cells can migrate to distant sites where they eventually differentiate into distinct cell types, including melanocytes, neurons, glial cells, adipocytes, and smooth muscle cells. NC cells that will later differentiate to melanocytes (pink) mainly migrate on the dorsolateral route (between somites and the ectoderm) towards the dermis; however, a subpopulation also migrates on the ventral route. During this phase of melanocytic development, SOX10 and PAX3 cooperate to activate the expression of MITF, the master regulator of the melanocytic lineage. (**D**) Migrating melanocytic precursors (melanoblasts, light pink) reach the dermis, start to proliferate, and pass the basement membrane to reach the epidermal-dermal junction. After entering the epidermis, the proliferation is even increased. Here, the cells distribute throughout the junctional epidermis and finally differentiate into pigment-producing melanocytes (brown). Orchestrated by MITF and its upstream regulators SOX10 and PAX3, a variety of enzymes, including tyrosinase and dopachrome tautomerase, are expressed that are essential for melanogenesis.

Altogether, a network of transcription factors tightly controls NC formation, NC cell migration, and melanocytic maturation (Figure 1). Insights into NC development and the impact of distinct transcription factors on induction, migration, and differentiation have been mostly gained in animal models such as mouse-, chick-, Xenopus-, and zebrafish embryos. 

PAX3 and msh homeobox 1 (MSX1), which are often termed NC-specific factors, are among the factors that are already expressed during the very early phases of NC development [29,30]. During this phase, the expression of early NC markers MSX1 and PAX3 is induced by bone morphogenetic protein (BMP) and WNT signaling [30,31,32,33]. During NC specification, MSX1 induces the expression of early NC markers SNAIL, SLUG (which are essential for epithelial-to-mesenchymal transition (EMT) of cells), forkhead box D3 (FOXD3), and PAX3 [31,34], highlighting its crucial role for NC induction [34]. However, when NC cells start to migrate, PAX3, MSX1, and FOXD3 are downregulated whereas SLUG expression is maintained [32]. Accordingly, other factors become essential for the survival of the migrating melanocytic precursors. SOX10 belongs to the HMG-box family of transcription factors and plays an essential role in the development of melanocytes and other NC cells [35,36]. SOX10 expression is initiated already in the very early phases of NC formation even before NC cell migration takes place, and expression is maintained throughout the migratory phase of melanocytic precursors [35,37,38]. It does not seem to be necessary for NC initiation [36]; however, SOX10 is crucial for the survival of migrating NC cells and specification of the melanocytic lineage as demonstrated in previous animal studies [39]. 

Another crucial factor required on the way from pluripotent NC cells to differentiated melanocytes is MITF. This transcription factor is among the first lineage-specific markers that are expressed by melanocytic precursors [21,40]. MITF is essential for the survival of melanocytic precursor cells during migration and is required for proliferation, lineage specification, and maturation. Besides, it regulates the expression of enzymes that are essential for melanin biosynthesis [41]. MITF is coded by a large gene spanning almost 230,000 bp on chromosome 3 [42]. Its genetic organization is quite complex as multiple transcription start sites (TSS) exist and alternative splicing also occurs, resulting in several isoforms [42]. The isoforms differ in the first exons that are called 1A, 1B1a, 1C, 1D, 1E, 1J, 1H, and 1Mc, and all of them are regulated by distinct promoters, whereas exons 2 to 9 containing the functional protein domains are shared between the distinct isoforms [42,43]. Melanocytes express the specific M-MITF isoform that is generated from the TSS in exon 1M [44]. Notably, SOX10 and PAX3 directly promote the expression of the melanocytic MITF isoform by binding it to adjacent sites in the MITF promoter [45,46]. In contrast to this, FOXD3 acts as a transcriptional repressor that directly impairs MITF expression [41,47], melanogenesis, and melanoblast migration [48]. Similar to SOX10 and PAX3, it is also expressed already before NC cell migration [49]. 

In this review, we will take a closer look at these main transcription factors that play a role both in the development of NC initiation, migration, and melanocyte formation and discuss their contribution to NC induction, NC cell migration, melanocyte development, melanoma formation, progression, and tumor plasticity.

## 3. Tumor Heterogeneity and Cellular Plasticity in Melanoma

Apart from its extraordinary embryonic origin, melanoma’s aggressiveness is also attributable to its vast intratumoral heterogeneity. This term describes a tumor that is composed of different tumor cell subpopulations expressing distinct gene signatures with various phenotypes. Heterogeneously composed tumors may adapt faster to environmental changes than homogenous tumors, resulting in drug resistance [50]. Intratumoral heterogeneity can be the result of coding mutations or transient epigenetic alterations involving histone modifications, DNA methylation, and chromatin remodeling [51,52]. The concept of tumor plasticity is also important in this context. It indicates that heterogeneity is not a one-way road but more likely a dynamic process allowing cells to switch back and forth between different phenotypic states (phenotype switching) instead of following a hierarchical model. Tumor heterogeneity also contributes to tumor progression through altered migratory and invasive properties [53]. Interestingly, genes defining the invasive phenotype and those defining a proliferative signature differ significantly and those states may be altered by anti-tumor treatments [54].

Multiple studies have demonstrated that melanoma is a rather heterogeneous tumor. For example, the H3K4 demethylase JARID1B has been identified as an epigenetic marker of a slow-cycling, drug-resistant subpopulation in melanoma, which has an increased capacity of self-renewal in vitro [55]. The fact that less than 5% of all cells expressed JARID1B demonstrates that the importance of such small subpopulations should not be underestimated and additionally that such subpopulations can essentially contribute to tumor progression [55]. Heterogeneity is a phenomenon observed in virtually all states of tumor progression, involving melanoma cells of the same tumor, between distinct metastases [56,57], and even in circulating tumor cells [58]. 

In the last few years, the development of less expensive and therefore widely available next-generation sequencing approaches has paved the way towards a better understanding of tumor heterogeneity [59]. In contrast to this, conventional bulk sequencing approaches are more suitable to identify the most dominant gene signature in a tumor [60]. However, single-cell RNA sequencing and spatial transcriptomic approaches are suitable to study tumor composition on a cellular level, leading to astonishing insights into intratumoral melanoma heterogeneity [56,60,61]. Therefore, we now also have a more precise picture of NC transcription factors and how they contribute to melanoma plasticity.

## 4. The Best from Both Worlds: Neural Crest Transcription Factors and Their Contribution to Melanoma Plasticity

Melanoma evolution is influenced by a variety of transcription factors. For example, runt-related transcription factor 2 (RUNX2) contributes to tumor progression by upregulating the expression of receptor tyrosine kinases and thereby mediating BRAFi resistance [62]. Additionally, other factors such as bromodomain PHD finger transcription factor (BPTF) [63], glioma-associated oncogene homolog 1 and 2 (GLI1, GLI2) [64], c-FOS, JunB proto-oncogene (JUNB), ETS proto-oncogene 2 (ETS2), and ETS variant transcription factor 1 (ETV1) [65] have been associated with melanoma progression and treatment resistance. 

Interestingly, transcription factors that are involved in NC development and melanocyte formation play a special role in this setting. These factors are also expressed in melanoma and have been studied extensively. Remarkably, these factors are also involved in melanoma plasticity and ultimately contribute to drug resistance.

### 4.1. MSX1: An “Early Bird” in NC Formation

MSX1 expression occurs in the very first phases of NC development [34,66]. As already discussed above, its expression decreases during the migratory phase, and differentiated melanocytes barely express MSX1 [32,67]. Little is known about its role in melanocytes and melanoma, but there is evidence that MSX1 promotes melanoma progression and induces phenotype switching [67]. Activation of Notch1 signaling is sufficient to reprogram differentiated melanocytes to multipotent NC stem cell (NCSC)-like cells [68]. These cells express high levels of MSX1, indicating that this factor mediates dedifferentiation and probably plays a role in phenotype switching of melanoma cells [68]. Heppt et al. demonstrated that ectopic expression of MSX1 reprograms melanocytes to a dedifferentiated NCSC-like state that is characterized by a loss of MITF expression, decreased pigmentation, expression of E-cadherin, increased expression of the neural crest marker p75, and the acquisition of stem cell-like properties [67]. These cells could be transdifferentiated to other NC-derived cell types as neurons, adipocytes, and smooth muscle cells, demonstrating their multipotent properties [67]. Similarly, MSX1 expression is sufficient to reprogram melanoma cells as well by triggering a phenotype switch [67]. Ectopic MSX1 expression in melanoma cells increased their ability to migrate, downregulated the expression of MITF, zinc finger E-box binding homeobox 2 (ZEB2), and E-cadherin, and increased the expression of WNT5A and zinc finger E-box binding homeobox 2 (ZEB1) instead, whereas MSX1 downregulation decreased migration and formation of liver metastases in a xenograft mouse model [67]. Further experiments showed that increased ZEB1 expression is most likely responsible for the MSX1-driven migratory phenotype [67]. Interestingly, MSX1 expression levels correlate with disease progression in melanoma patients, and patients with high MSX1 expression levels have a poorer OS [67]. 

Altogether, these results indicate that MSX1 contributes to tumor plasticity by inducing a phenotype switch towards an invasive and metastasis-promoting phenotype, which is associated with an unfavorable prognosis.

### 4.2. MITF and Its Upstream Regulators SOX10, PAX3, and FOXD3

MITF is by far the most intensively studied of these factors regarding melanoma formation, progression, and plasticity due to its prominent role in melanocyte development and melanogenesis. It is difficult to separate its function in melanoma plasticity entirely from its various upstream regulators including SOX10, PAX3, and FOXD3 among others, as they are tightly connected. Therefore, these factors and their contribution to melanoma plasticity will now be discussed within the following sections of this manuscript.

Due to its central role in melanogenesis, MITF in melanoma has been extensively studied so far. About 20% of metastatic melanomas harbor MITF gene amplifications [69,70], and MITF expression is detected in the majority of primary melanomas (except for desmoplastic melanoma) [71]. Interestingly, about 50% of relapsed melanoma express reduced levels of MITF [72]. Additionally, MITF^E318K^ mutations affecting SUMOylation of the protein and thereby increasing its transcriptional activity have been linked to familial melanoma [73]. Notably, genetic aberrations, including amplifications as well as mutations leading to alterations of evolutionarily conserved amino acids in functional domains such as the basic helix-loop-helix and activation domains, were detected in 16% of metastatic melanoma samples [74]. MITF can have both tumor-promoting and suppressive features. Thus, whether MITF promotes or suppresses melanoma remains ambiguous. On the one hand, MITF is amplified in melanoma metastases and patients with MITF amplifications have poorer survival, indicating that MITF may act as an oncogene [69]. MITF directly regulates genes that are required for DNA replication and cell cycle progression, including *CDK2* [75], *TERT*, *LIG1*, *CCNB1*, *CCNF*, and *CCND1* [76]. Additionally, short-term MITF depletion downregulates the diaphanous-related formin 1 (DIA1), leading to a p27-dependent cell cycle arrest and increases rho-associated coiled-coil containing protein kinase (ROCK)-dependent invasiveness of the cells, indicating a tumor-promoting role of MITF [77]. On the other hand, increased MITF expression decreased the invasiveness of melanoma cells by reducing DIA1 expression, which controls actin polymerization and is therefore involved in cytoskeletal reorganization [77]. Giuliano et al. demonstrated that long-term depletion of MITF induces senescence via DNA damage response in a p53-dependent manner [78]. MITF can promote cell cycle arrest by upregulating the cyclin-dependent kinase inhibitors p21^Cip1^ and p16^INK4a^ [79,80].

These data indicate that MITF promotes distinct functions depending on its expression levels. Based on their studies, Carreira et al. have proposed a model suggesting that either proliferation, invasion, or differentiation are promoted depending on the MITF expression levels [53,77]. According to this model, low MITF expression is associated with a stem-cell-like, G1-arrested but invasive phenotype; intermediate MITF levels are associated with proliferating cells, and high MITF expression with a differentiated but G1-arrested phenotype (“rheostat model”) [77]. 

Expression of the receptor tyrosine kinase AXL often inversely correlates with the MITF expression levels. The invasive MITF^low^ AXL^high^ phenotype was first described by Sensi et al. [81] and has been previously linked to BRAF and MEK inhibitor (BRAFi, MEKi) resistance [82,83], as AXL may transmit survival signals irrespective of MAP kinase (MAPK) signaling [60]. Being a direct upstream regulator of MITF, SOX10 has also been associated with the proliferative MITF^high^ phenotype [84]. However, SOX10 can also promote invasion by upregulating its direct target genes melanoma inhibitory activity (MIA) and peripheral myelin protein 2 (PMP2) [85,86]. Interestingly, MITF^low^ cells are resistant to anoikis, which is cell death after detachment and clearly beneficial for circulating melanoma cells [87]. Maurus et al. could show that this resistance was mediated by the transcription factor FOSL1, which downregulates MITF in an HMGA1-dependent manner [87].

#### 4.2.1. MITF, Heterogeneity and Plasticity

**Figure 2:**
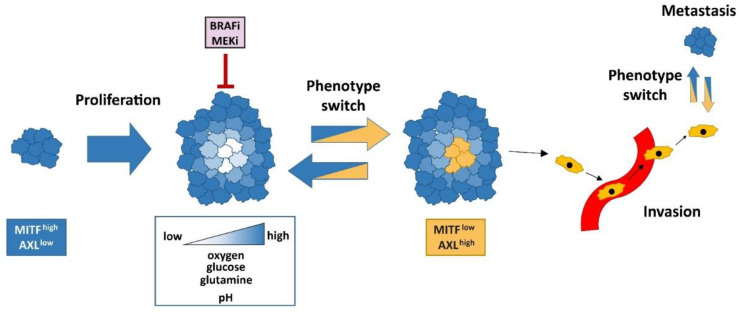
Phenotype switch in melanoma. Cells expressing high levels of MITF and low levels of the receptor tyrosine kinase AXL are characterized by high proliferative capacity and thereby promote tumor growth. As a result, melanoma cells located at the center of the growing tumor often experience a decrease in oxygen, glucose, and glutamine levels, which also contributes to acidic extracellular pH levels. These changes of the tumor microenvironment can trigger a switch from a MITF^high^ AXL^low^ to a MITF^low^ and AXL^high^ phenotype. Cells of this phenotype barely proliferate but they have an increased invasive capacity and promote the formation of metastases. Similarly, systemic therapies inhibiting BRAF and MEK (BRAFi, MEKi) can also promote phenotype switching towards a MITF^low^ AXL^high^ phenotype.

MITF plays a central role in mediating intratumoral heterogeneity, plasticity, and phenotype switching in melanoma (Figure 2) [88]. One of the first hints pointing towards the important role of MITF in phenotype switching was observed by Hoek et al. when they established xenografts of MITF^low^ and MITF^high^ expressing melanoma cell lines and observed that these tumors expressed both proliferative and invasive transcriptional signatures [89]. Additionally, MITF expression of the xenograft tumors was independent of the initial MITF expression status of the injected cell line, indicating that melanoma cells can switch dynamically between different phenotypes [89]. Their data were in line with the model proposed by Carreira et al. [77], demonstrating the existence of a proliferative, MITF^high^ phenotype and an invasive, MITF^low^ phenotype [89]. It is interesting to note that the BRAF mutational status does not seem to have an impact on the phenotype-specific invasive and proliferative gene expression profiles, respectively [90]. 

Tirosh et al. sequenced more than 4500 single cells of 19 melanoma patients and discovered that a subpopulation of treatment-resistant cells was already present before the treatment [56]. They also observed a transcriptional heterogeneity of melanoma cells, which was associated with the cell cycle, drug-resistance program, and the spatial distribution inside the tumor [56]. All tumors harbored two cell populations with distinct transcriptional phenotypes, namely, MITF^high^ AXL^low^ and MITF^low^ AXL^high^, although they could be classified on the bulk tumor level as either MITF^high^ or AXL^high^ [56]. In vitro, BRAFi and MEKi led to an increase in AXL-positive cells in the cell lines that had a low baseline AXL-positive fraction, whereas those with a higher number of AXL-positive cells per se showed only small or no changes [56]. This important work also demonstrated that both invasive and proliferative signatures existed within the tumors at the same time, highlighting intratumoral heterogeneity of melanoma [56]. Besides, the stroma of MITF^high^ tumors consisted of a smaller number of cancer-associated fibroblasts and the tumors showed a higher T cell infiltration, suggesting that the composition of the tumor microenvironment (TME) is different depending on MITF expression levels [56]. 

Interestingly, the phenomenon of intratumoral heterogeneity regarding MITF expression levels is not restricted to tumors only but is also found in vitro. Ennen et al. observed significant heterogeneity of single cells regarding MITF expression levels and gene signatures in a MITF-positive cell line [91]. Furthermore, some cells with high MITF expression levels had an “invasive” gene signature [91], indicating great intratumoral heterogeneity. However, a rather low heterogeneity regarding gene signatures was observed in an AXL/WNT5A-positive cell line that did not express MITF [91].

Another work of this group investigating intra- and intertumoral heterogeneity in melanoma by single-cell RNA sequencing confirmed previous findings of MITF^high^ and MITF^low^ subpopulations in melanoma samples [92]. Heterogeneity was observed in both primary tumors and cutaneous metastases where MITF and SOX10 were identified as markers of the proliferative MITF^high^ subpopulations. SOX10 and PAX3 expression levels correlated best in this study [92]. Remarkably, Ennen et al. identified a third distinct subpopulation that simultaneously expressed genes of the MITF^high^ as well as MITF^low^ populations [92]. This indicates that there may be a new cell phenotype apart from MITF^high^ and MITF^low^. 

Dynamic MITF expression is highly advantageous for melanoma, and distinct subpopulations cooperate to promote invasiveness. For example, poorly invasive MITF^high^ melanoma cells can cooperate with highly invasive MITF^low^ cells to enable invasion, and both phenotypes are present at the invasive front, indicating that MITF expression does not necessarily have to be downregulated for successful melanoma invasion [93]. Thus, it remains unclear if a phenotype switch is even necessary to promote invasion and both MITF^high^ and MITF^low^ cells can benefit from each other.

#### 4.2.2. What Alters the Expression of MITF to Promote Cellular Plasticity?

**Figure 3:**
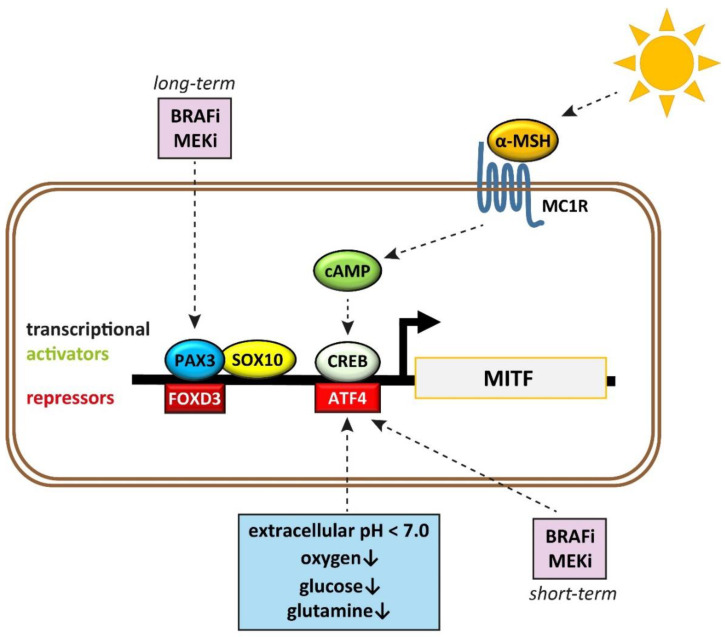
Transcriptional regulators of MITF in melanocytes and melanoma. MITF expression is induced by SOX10, PAX3, and CREB. UV radiation induces α-MSH expression, which binds to its receptor MC1R. This leads to an increase of cAMP and subsequently activates CREB. In contrast, low levels of oxygen, a lack of nutrients, and an acidic extracellular pH can decrease MITF transcription via ATF4 that acts as a transcriptional repressor. BRAF and MEK inhibition (BRAFi, MEKi) can either repress or stimulate MITF expression depending on the treatment duration.

Given the fact that MITF plays such a prominent role, it is interesting to study what triggers alterations in MITF expression and therefore drives MITF-dependent cellular plasticity. Here, different intrinsic and extrinsic factors are involved (Figure 3). The phenotype switch towards an invasive and dedifferentiated phenotype can be triggered by secreted factors, including tumor necrosis factor (TNF), transforming growth factor- β (TGF-β), and WNT5A [60,89,94]. However, MITF has the dominant role in the switching process as the transition of the proliferative state into the invasive state only occurs when MITF is suppressed [77].

MITF expression can be altered by autocrine and paracrine TGF-β signaling, for example, TGF-β signaling downregulates MITF in melanocyte stem cells [95]. Additionally, Hoek et al. demonstrated that low MITF gene expression profiles were associated with high TGF-β signaling in melanoma [84]. TGF-β signaling induced hypopigmentation and cell motility in a melanoma xenograft model [96]. Taken together, these results indicate that TGF-β may be one of the factors mediating phenotype switching from a proliferative to a more invasive phenotype. Besides, other pathways such as p38 MAP kinase [97], Notch (reviewed in [53]), and β-catenin, which directly transactivate the M-MITF promoter by binding to a LEF-1 binding site [98], can also directly interact with MITF [99]. Therefore, these pathways are also likely to be involved in modulating MITF levels and thereby contributing to phenotype switching and tumor heterogeneity in the end. Interestingly, the TME and stiffness of the extracellular matrix (ECM) have been shown to impact MITF expression levels in melanoma, and TGF-β is also partly involved in this regulation [100]. A stiffer ECM with increased collagen levels induces proliferation and differentiation of melanoma cells via Yes1-associated transcriptional regulator (YAP) and PAX3, which interact and thereby promote MITF expression [100]. In line with this, gene expression of MITF target genes seemed to correlate with poorer survival in melanoma patients with high-collagen tumors [100]. However, TGF-β which can be secreted by cancer-associated fibroblasts can trigger a SMAD- and YAP/TEAD-driven transcriptional program, leading to a downregulation of MITF expression as well as its target genes [100]. Moreover, MITF can shape the TME itself as it represses the expression of several genes involved in ECM, EMT, and focal adhesion by binding to its promoters and thereby inhibiting the transcription [101].

Other important environmental factors that repress MITF expression include hypoxia, starvation, and extracellular acidosis. Low levels of oxygen reduce MITF expression in melanoma in an indirect hypoxia-inducible factor 1 subunit alpha (HIF-1α)-dependent manner, thereby promoting the invasive, metastatic phenotype [102,103]. In detail, this effect is mediated via the transcription factor class B basic helix-loop-helix protein 2 (BHLHB2), which is upregulated upon hypoxic conditions and can bind to the MITF promoter, repressing its transcription [102]. Glucose restriction increases the production of reactive oxygen species, which causes upregulation of activating transcription factor 4 (ATF4) that suppresses MITF expression [104]. Similarly, prolonged culturing of melanoma cells in media lacking the amino acid glutamine leads to decreased MITF expression levels also mediated via upregulation of ATF4 [105]. B16 mouse melanoma cells also formed more lung metastasis in a tail vein injection model with immunocompetent C57BL/6 mice when cultured in glutamine-free media prior to injection [105]. Recently, Böhme et al. discovered that extracellular acidosis inhibits eIF2α and leads to an activation of ATF4 expression, resulting in downregulation of MITF and upregulation of AXL [106]. Acidic pH levels between 5.5 and 7.0 are frequently observed in tumors as a result of increased glycolysis (“Warburg effect”), glutamine consumption, and high activity of the pentose phosphate and HIF pathways (reviewed in [107]). Altogether, these results indicate that an insufficient supply of oxygen, nutrients, and an acidic TME may induce a switch towards the invasive MITF^low^ AXL^high^ phenotype and thereby drive tumor progression [104,105]. 

PAX3 is also frequently expressed in primary melanoma and to a lesser extent in benign nevi [108]. Another work by Smith et al. demonstrated that BRAF can control MITF expression levels by acting on the transcription factors brain-2 (BRN2) and PAX3 [109]. They observed that PAX3 expression correlates with MITF expression levels and discovered that expression levels of the transcription factor BRN2 were inversely correlated with PAX3 and MITF expression and BRN2 and PAX3 interacted in vitro to control MITF transcription. Based on their findings, they developed a BRN2-PAX3 rheostat model that explains how MITF expression is controlled in BRAF^V600E^ mutant melanoma. According to this model, low ERK phosphorylation (pERK), indicating low MAPK pathway activity, promotes PAX3-mediated MITF expression, whereas high pERK promotes high BRN2 expression and represses PAX3-mediated MITF expression, leading to a MITF^low^ phenotype [109]. 

Furthermore, MITF expression is also epigenetically controlled via DNA methylation [110]. In cells with an invasive phenotype, the promoters of MITF and several of its target genes are silenced via hypermethylation [110]. Thus, promoting the transition of the invasive into a proliferative state in vitro is more challenging than the other way round as MITF overexpression is obviously not sufficient to achieve this transition [60].

## 5. Plasticity as a Reason for Treatment Resistance

The development of BRAFi has changed the treatment spectrum of metastatic melanoma, resulting in rapid initial responses. Nevertheless, it has become evident that drug resistance is a common phenomenon [1]. Intrinsic resistance mechanisms lead to primary treatment failure in about 15% of patients and, additionally, the majority of patients experience disease progression after a few months, although they had initially responded to BRAF [111]. Intratumoral heterogeneity is one factor that significantly contributes to the treatment failure of MAPK inhibition. Phenotypic alterations can be both permanent and reversible, as in the case of a phenotype switch [52,53]. On the one hand, resistance to BRAFi is caused by mutations affecting NRAS, and MEK activators like CRAF or MEK, leading to reactivation of MAPK signaling or upregulation of PI3K/AKT signaling [112]. On the other hand, transcriptomic changes without mutations are common. Phenotype switching is thought to be triggered by changes in the TME when new biological properties are required [50,53]. Indeed, melanoma plasticity significantly contributes to the treatment failure of targeted therapies affecting MAPK signaling [113]. Recent evidence suggests that melanoma plasticity also redounds to failure of immune checkpoint blockade [114]. Tumors of patients that did not respond to anti-PD-1 immune checkpoint blockade had significantly higher expression levels of the receptor tyrosine kinase AXL compared to tumors of responders [114].

### 5.1. Targeted Therapy

Several studies investigating the role of NC transcription factors in melanoma plasticity have been published. MITF seems to play an important role in establishing resistance against BRAF and MEK inhibition, especially during the early phases of acquired drug tolerance [72]. Long-term inhibition of the MAPK pathway increases the expression of MITF via its upstream transcriptional regulator PAX3 [72]. The question of whether there is a connection between MITF and BRAFi resistance is not easy to answer. On the one hand, high MITF expression due to gene amplification has been associated with BRAF resistance [115]. On the other hand, the slow-cycling MITF^low^ expressing phenotype is also resistant to MAPK pathway inhibition [83].

Shaffer et al. treated naïve melanoma cells with BRAFi and showed that BRAFi-resistant colonies arose from single cells, which proliferated without BRAFi treatment, indicating that these resistant cells were not part of a dormant cell population [116]. They also showed that the drug-resistant phenotype was not heritable or caused by mutations but transient and reversible instead [116]. Within the first weeks of BRAF inhibition, ATAC-seq revealed a loss of accessible transcription factor binding sites, followed by an increase in accessible sites. Interestingly, the loss of SOX10 binding seemed to be a major contributor to the initial peak loss. On the other hand, the gain of sites seems to be attributable to the activation of TEAD, Jun/AP-1, and other signaling pathways [116]. These results indicate that dedifferentiation followed by activation of new signaling pathways is required to establish drug resistance [116].

Another example of cooperation of distinct subpopulations was previously described by Smith et al. [117]. Endothelin 1 (EDN1), which is secreted by MITF^high^ cells upon exposure to BRAFi, promotes the proliferation of AXL^high^ cells via endothelin receptor type A (EDNRA) signaling in a paracrine manner [117]. Thereby, MITF^high^ cells can promote the expansion of the AXL^high^ subpopulation upon BRAFi via protein kinase C (PKC) and CRAF to circumvent MAPK pathway inhibition [117]. Endothelin receptors (EDNRs) are expressed in both MITF^high^ AXL^low^ and MITF^low^ AXL^high^ cells [117]; thus, inhibiting EDNRs may be a promising alternative to overcome BRAFi resistance and circumvent the problem of heterogeneous MITF expression in melanoma. 

Furthermore, ATF4 that suppresses MITF expression as described above plays also a role in acquired resistance to BRAFi and MEKi. Recently, Yang et al. discovered that ATF4 stress signaling mediates to a rapid escape already within the first days after initiating MAPK pathway inhibition [118]. Initially, melanoma cells expressed higher levels of MITF when exposed to BRAFi and MEKi, which was in line with a previous report of Smith et al. [72], followed by dedifferentiation and a slow-cycling phenotype marked by a decrease of MITF and an increase in nerve growth factor receptor (NGFR) expression [118]. However, single-cell RNA sequencing revealed that a MITF^low^ AXL^high^ subpopulation was already present during this first phase of acquiring drug resistance [118]. Knockdown experiments showed that the cells were dependent on ATF4 expression to escape MAPK pathway inhibition [118].

Another factor recently identified to regulate MITF and SOX10 in melanoma is the helix-loop-helix transcription factor inhibitor of DNA binding 3 (ID3) [119], which acts as a transcriptional repressor [120]. It plays a role in cell cycle progression and is required for the survival of NC progenitors during embryogenesis [121]. Interestingly, ID3 is upregulated in BRAFi-resistant melanoma compared to pretreatment [119]. In vitro, BRAFi also led to an increase in ID3 expression accompanied by a decrease in the expression of MITF, SOX10, and other differentiation markers [119]. ID3 overexpression was also associated with a higher migratory capacity, whereas ID3 knockdown resulted in a strong increase of SOX10 and MITF expression, indicating that ID3 acts as a transcriptional repressor of SOX10 and MITF expression, thereby promoting a drug-resistant phenotype [119]. However, Sachindra et al. did not investigate whether ID3 binds directly to the promoters of MITF and SOX10. 

SOX10 also plays an important role in mediating drug resistance to BRAF inhibition. Downregulation of SOX10 activates TGF-β signaling, resulting in an upregulation of epidermal growth factor receptor (EGFR) and platelet-derived growth factor receptor beta (PDGFRB) and a slow-cycling phenotype, thereby contribute to resistance to BRAF and MEK inhibition by providing survival signals independently of the MAPK pathway [122]. The percentage of SOX10^low^ EGFR^high^ melanoma cells displaying a slow-cycling phenotype increases after treatment with BRAFi and MEKi [122]. This highlights that SOX10 expression also mediates cellular plasticity in melanoma. Another study investigating long-term exposure to BRAFi in melanoma observed that cells expressing high levels of EGFR showed increased cell migration, a high ERK activity suggesting resistance to BRAFi, and decreased sensitivity to EGFR inhibition with erlotinib [123]. Interestingly, EGFR^high^ cells also expressed higher levels of PD-L1, suggesting that immune checkpoint blockade may be a suitable alternative to MAPK pathway inhibition [123].

In melanoma, FOXD3 can directly upregulate PAX3 by binding to its promoter [124]. FOXD3 is suppressed by BRAF, and it acts as a cell cycle repressor by upregulating p21^Cip1^ [125]. Interestingly, FOXD3 upregulates Erb-B2 receptor tyrosine kinase 3 (ERBB3) expression and thereby mediates resistance to BRAFi and MEKi [126]. 

Although several studies linked MITF, SOX10, and other factors to BRAFi and MEKi resistance as summarized above, there is evidence that the development of drug resistance to targeted therapies seems to be even more complex. Hartman et al. recently detected distinct genetic and non-genetic alterations, including varying expression levels of MITF, NGFR, and SOX10 in BRAFi- and MEKi-resistant patient-derived cell lines [127]. This indicates that resistance to targeted therapies develops in a patient- and drug-dependent manner and may not be limited to only a few key factors [127].

### 5.2. Immunotherapy

There is growing evidence that melanoma plasticity also plays a role in resistance to immunotherapy. A dedifferentiated cell phenotype expressing mesenchymal transition genes, including AXL and WNT5A, has been linked to resistance to PD-1 blockade in melanoma patients [114]. These results indicate that the slow-cycling, invasive MITF^low^ AXL^high^ phenotype mediates some kind of “cross-resistance” to immune checkpoint blockade targeting PD-1 [114]. Additionally, Landsberg et al. demonstrated in a mouse model that melanoma cells can switch between a differentiated and dedifferentiated state to acquire resistance to adoptive cell transfer in response to inflammatory stimuli [128]. In particular, they were able to identify TNF-α as the crucial factor responsible for the transient dedifferentiation in response to T cell therapy [128]. Recently, an interesting study elucidated the role of MITF in resistance to innate immunity in melanoma and identified it as a transcriptional regulator of ADAM metallopeptidase domain 10 (ADAM10) [129]. ADAM10 is a membrane-anchored metalloprotease [130] that cleaves the natural killer group 2, member D (NKG2D) ligands MHC class I polypeptide-related sequence A (MICA), and to a lesser extend MHC class I polypeptide-related sequence B (MICB) [131,132]. Thus, MITF-dependent upregulation of ADAM10 impairs melanoma cell recognition by natural killer (NK) cells, resulting in the escape of MITF^high^ expressing cells [129].

The chemokine expression pattern in melanoma cells and immune cell attraction is also affected by MITF, indicating the immune cell infiltration may depend on [133]. This is interesting as “cold tumors” displaying a low number of infiltrating immune cells are less susceptible to immunotherapies targeting T cells as immune checkpoint blockade for instance [134,135]. Regarding human melanoma cell lines, decreased expression levels of MITF promoted immune cell migration of CD14+ monocytic cells and CD56+ NK cells, indicating that MITF^low^ cells may attract these cells more efficiently, whereas attraction of B cells and T cells was barely influenced by MITF expression [133]. Furthermore, an increased expression of the chemokines CCL2, CXCL1, CCL15, and CCL19 was observed after MITF knockdown [133]. 

The expression of PD-L1 and other factors involved in immune checkpoints has an impact on the success of immunotherapy. MITF can trigger lysosomal degradation of PD-L1 in melanoma cells [136]. Recently, FOXD3 was also identified as a regulator of V-domain immunoglobulin suppressor of T cell activation (VISTA) expression in melanoma [137]. VISTA is an immune checkpoint protein, and its role and expression in melanoma have been barely characterized before. VISTA is expressed in melanoma, and VISTA overexpression does not have an impact on in vitro cell proliferation, wound healing, or invasion assays, but it promotes tumor onset in immunocompetent mice, and in VISTA-expressing tumors a higher number of immunosuppressive T_regs_ was present although infiltration and activation of CD4+ and CD8+ T cells was not altered [137]. Interestingly, FOXD3 expression in response to BRAF inhibition is sufficient to reduce VISTA expression of melanoma cells [137]. Given the fact that cell differentiation is significantly influenced by MITF, these studies highlight how differentiation contributes to immune checkpoint blockade failure.

## 6. Conclusions

Cutaneous melanoma is characterized by high intratumoral heterogeneity. The diverse roles of MITF, its upstream regulators, and MSX1 highlight that factors expressed during NC cell and melanocytic development contribute to melanoma cell plasticity. Understanding this delicate network and how it contributes to intratumoral heterogeneity, development of drug resistance, and ultimately treatment failure may also pave the way towards new treatment strategies for resistant melanoma.
